# Situational Use of Child Restraint Systems and Carpooling Behaviors in Parents and Caregivers

**DOI:** 10.3390/ijerph15081788

**Published:** 2018-08-20

**Authors:** Catherine C. McDonald, Erin Kennedy, Linda Fleisher, Mark R. Zonfrillo

**Affiliations:** 1 Department of Family and Community Health, School of Nursing, University of Pennsylvania, Philadelphia, PA 19104, USA; erinken@nursing.upenn.edu; 2 Department of Pediatrics, Perelman School of Medicine, University of Pennsylvania, Philadelphia, PA 19104, USA; FleisherL@email.chop.edu; 3 Center for Injury Research and Prevention, Children’s Hospital of Philadelphia, Philadelphia, PA 19104, USA; 4 Penn Injury Science Center, University of Pennsylvania, Philadelphia, PA 19104, USA; 5 Department of Emergency Medicine, Alpert Medical School of Brown University and Hasbro Children’s Hospital, Providence, RI 02903, USA; zonfrillo@brown.edu

**Keywords:** booster seat, carpooling, car seat, children, child restraint system, parent, seat belt

## Abstract

Suboptimal compliance with child restraint system (CRS) recommendations can increase risk for injury or death in a motor vehicle crash. The purpose of this study was to examine scenarios associated with incomplete CRS use and non-use in children ages 4–10 years. We used a cross-sectional online survey with a convenience sample of parent/caregivers from the United States, age ≥18 years, with a child age 4–10 years in their home, who could read and spoke English, and drove child ≥6 times in previous three months. We used descriptive statistics and Mann-Whitney U to describe and compare the distribution of responses to situational use of CRSs among car seat users and booster seat users. We also used descriptive statistics and the Mann-Whitney U to describe and compare the distribution of responses to carpooling items among booster seat users and non-booster seat users. There were significant differences among those who reported most often using booster seats (n = 282) and car seats (n = 127) in situations involving rental cars, driving just around the corner, car too crowded to fit the CRS, not enough CRSs in the vehicle, the CRS is missing from the car, or the child is in someone else’s car without a CRS (*p* < 0.05). Among those who reported most often using booster seats and who carpooled other children (n = 159), 71.7% (n = 114) always used a booster seat for their own child. When carpooling other children, booster seat users were significantly more likely to use booster seats for other children ages 4–10 than the non-booster seat users (*p* < 0.01). Continued education and programs surrounding CRS use is critical, particularly for children who should be in booster seats.

## 1. Introduction

Motor vehicle crashes remain a leading cause of injury and death in children. Suboptimal compliance with child restraint system (CRS) recommendations [1,2] can increase risk for injury or death if a crash does occur [3,4,5]. After children grow out of harness systems, booster seats help to adequately restrain children in the years they are not yet ready for the seat belts outfitted in vehicles. For example, without a booster seat, the rear seats of most vehicles are unlikely to provide good lap belt fit for up to 75% of children ages 6–12, and the shoulder belt fit is outside the target range for 40% of children [6]. 

Restraint use declines with age among children. The majority of infants are restrained at rates of approximately 98%, children ages 1–3 years at approximately 95%, and children ages 4–7 years old at approximately 85% [7]. The 2015 National Survey of the Use of Boosters Seats indicate that over 1/4 of children ages 4–7 years old prematurely graduate out of booster seats to seat belts, with an additional 10% who are unrestrained [8]. Even though we may have seen improvements in CRS use in the United States (U.S.), the percentage of children who are unrestrained or restrained in a way that is discordant with their age and size still places some children at risk for injury or death.

Factors associated with booster seat use and misuse may include lack of knowledge [9], lack of self-efficacy [10], cost, and need to accommodate other children in a vehicle [11]. However, a further understanding of the situations in which parents and caregivers inconsistently use CRSs is needed to determine modifications to education, technology, or policy may that increase compliance related to booster seat use in children. Given advancing technology in vehicles and CRSs, as well as changes in transportation options [12], continued understanding of parents’ behavior may lead to improved prevention efforts targeting high-risk groups. The purpose of this research study was to examine scenarios that are associated with incomplete CRS use or non-use in children ages 4–10 years.

## 2. Materials and Methods

We carried out a cross-sectional online survey with a convenience sample in the U.S. to collect data on parents’ and caregivers’ utilization of CRSs. Details of the survey methods are reported elsewhere [13]. Briefly, participants completed a short six-item screener to determine eligibility for a longer survey. Our inclusion criteria were as follows: Participant ≥ age 18 years; could read and spoke English; parent or routine caregiver of child in their home between ages 4–10 years; and had driven his or her oldest child between ages 4–10 years at least six times in the past three months. Recruitment occurred via TurkPrime, an online crowdsourcing platform targeting participant enrollment for academic research purposes from Mechanical Turk (mTurk). Behavioral science surveys have used the mTurk and TurkPrime platforms as a way to recruit participants [14,15,16,17]. Participants from these platforms are often more educated, less diverse and have a higher income than a nationally representative sample [18].

Individuals in TurkPrime who clicked on study recruitment materials were directed to a six-item screener in Qualtrics (Qualtrics, Provo, UT, USA). The six-item screener was constructed to collect data on eligibility and included questions on whether they were a parent or caregiver of one or more children in their home, gender, age, ability to read and speak English, age of child (ren), and whether they drove the oldest child between the age of 4–10 years six or more times in the past three months. Participants were compensated $0.20 (US) for the screener completion, whether or not they were eligible for the longer survey. Participants who did not meet criteria were informed they were not eligible for the longer survey and thanked for their time. Participants who met eligibility criteria were given study information, provided an online consent document and instructions for the longer survey about driving behaviors and child passenger safety practices in Qualtrics. Consent was obtained by clicking “yes” to the question “Do you agree that you have read and understand the information above and you consent to taking the survey?”, and those that consented were directed to the longer survey.

Participants were instructed to answer questions in reference to their oldest child between the ages of 4–10 years. Participants whose oldest child between ages 4–10 years most frequently used a car seat were directed to a survey where car seats were the referred type of CRS. Participants whose oldest child most frequently used a booster seat, seat belt, or none of the above were directed to a survey where booster seats were the referred type of CRS. Participants who completed the longer survey were compensated an additional $2.00. Data collection occurred from 6 March 2017–27 April 2017, with survey batches deployed so that up to 1500 participants in one batch could complete the screener. Participant data were anonymous. This study received exempt status from the University of Pennsylvania Institutional Review Board.

### 2.1. Demographic Data

We collected self-report data of age group, gender, child age, income, state of residence, race, and ethnicity. State of residence was recoded to region (North, South, East, and West).

### 2.2. CRS Situations Use

Participants were also asked to indicate “In the last 3 months, which of the following did you most often use for your oldest child between ages 4–10 years when you drove him/her?” Response options included: Car seat; Booster seat; Seat belt; and None of the above. Participants were also asked to estimate the height of their oldest child between ages 4–10 years. Response options included less than 38 inches (in) (96.5 centimeters (cm)), 38–47 in (96.5–119.4 cm), 48–56 in (121.9–142.2 cm), 57 in (144.8 cm) or more, and I do not know.

We modified items from Zonfrillo and colleagues [19] to collect data on CRS situational use. The modification included querying a three-month period and adding a “Neutral” option to the Likert scale options.

Participants were asked to respond to items about CRS use in reference to their oldest child between ages 4–10 years and the respective type of CRS used (car seat was the referred type for the car sear users and booster seat was the referred type for the booster sear, seat belt or none of the above users). The survey items included: “I would allow my oldest child between ages 4–10 years to ride in a car not fully buckled in their CRS when…” “My child is asleep when put in the car,” “My child is fussy and complaining about the CRS,” “My child is embarrassed about using a CRS because his/her friends do not use one,” “I am not driving far at all, just around the corner,” “There are too many people in the car to fit the CRS,” “There are not enough CRS seats for the number of children,” “The CRS is not in the car for some reason,” “It is a reward for my child,” “Traveling overnight and my child cannot sleep in the CRS,” “On vacation,” “My child keeps climbing out of the CRS,” “Someone is holding my child,” “Riding in a taxi cab or car sharing service (for example, Uber, Lyft),” “In a rental car,” “I am in a rush and do not have time,” “My child is in someone else’s car that does not have a CRS,” and “In a grandparent’s car.” Response options included Strongly Agree, Somewhat agree, Neutral, Somewhat Disagree, and Strongly Disagree.

### 2.3. Carpooling Behaviors

For those that indicated in the initial items that they used a booster seat, seat belt or none of the above, participants were also presented with items on carpooling behaviors, modified from Macy and colleagues [10]. In order to identify carpooling behaviors, the following items were used: “How often, in the last 3 months, did you transport children other than your own?” and “How often, in the last 3 months, was your child transported in a vehicle (carpooling where someone else was driving) other than your own?” Response options included 3 or more times per week, 1–2 times per week, Less than once per week, and Never.

For participants that indicated they transported other children, data from the following items were also examined and referred to the last three months: “When I carpool, I have all 4-to 10-year-old children ride in booster seats” and “My child rides in a booster seat when I am driving his or her friends who do not use booster seats.” For those that indicated their child was transported by others, data from the following items were also examined: “I ask other people to have my child use a booster seat when they are driving him/her” and “I have given other people a booster seat to use for my child when they are driving him/her.” Response options included Rarely/Never, Sometimes, Most of the time, and Always.

### 2.4. Survey Attention Check

We used an attention check presented to participants when they completed approximately 70% of the survey, modified from Oppenheimer [20] and used previously in studies using mTurk [21]. Respondents were presented with an item: “What sport do you like to play/watch?” The responses included four sports as options and the option “Other.” Above the item was a block of instructions for respondents to choose the “Other” option and write “I read the instructions” in the corresponding text box.

### 2.5. Analysis

Participants were excluded from analyses if they failed the survey attention check or reported inconsistent answers on CRS use or driving frequency (e.g., report in one item they typically used a booster seat and then in another item they never use one). Additional measures were taken to examine the validity of the data, including gender concordance of answers and age concordance of participant and child between the screener and longer survey. Supplementary Figure S1 outlines the derivation of the overall analytic sample (n = 783).

*Situational Use of CRS*: Inclusion criterion for the analysis of situational use of CRS was child height of 38–56 in (96.5–142.2 cm). Further inclusion criterion was parent/caregiver response to the question “In the last 3 months, which of the following did you most often use for your oldest child between ages 4–10 years when you drove him/her?” that they most often used car seats or booster seats for their oldest child age 4–10 years. Supplementary Figure S2 outlines the sample for the situational use of CRS analysis (n = 409).

We used percentages to describe the distribution of responses to the Likert Scale categories (Strongly Agree, Somewhat Agree, Neutral, Disagree, Strongly Disagree) for each item of situational use among the car seat users and booster seat users. We examined the distribution of responses using a Mann-Whitney U for car seat users and booster seat users on situational use of their CRS.

*Carpooling Behaviors*: As described, only parents/caregivers who reported most often using a booster seat, seat belt, or none of the above were presented with items regarding carpooling. Inclusion criterion for this analysis of carpooling behaviors was child height of 38–56 in (96.5–142.2 cm). Further, we included participants who endorsed carpooling (transporting other children or having own child being transported by another). Participants were categorized for carpooling behavior analysis as booster seat users and non-booster seat users (seat belt/none of the above). Supplementary Figures S3 and S4 outlines the carpooling behaviors sample for this analysis for transporting other children and having own child be transported by another.

We used percentages to describe the distribution of responses to the Likert Scale categories (Always, Most of the Time, Rarely, Sometimes, Never) for each carpooling item. We used a Mann-Whitney U to examine the differences among booster seat users and non-booster seat users for the item “When I carpool, I have all 4–10 year olds ride in a booster seat.”

## 3. Results

### 3.1. Situational Use

There were 409 participants for the situational use of CRS analysis (Supplementary Figure S2). Table 1 displays the demographic characteristics for the sample by car seat and booster seat use. Participants in the overall sample were 89.2% (n = 365) White, 3.7% (n = 15) Asian, 3.4% (n = 14) more than one race, 2.9% Black/African American, and 0.7% (n = 3) American Indian or Alaska Native. No one reported as Native Hawaiian or Other Pacific Islanders.

**Figure 1:**
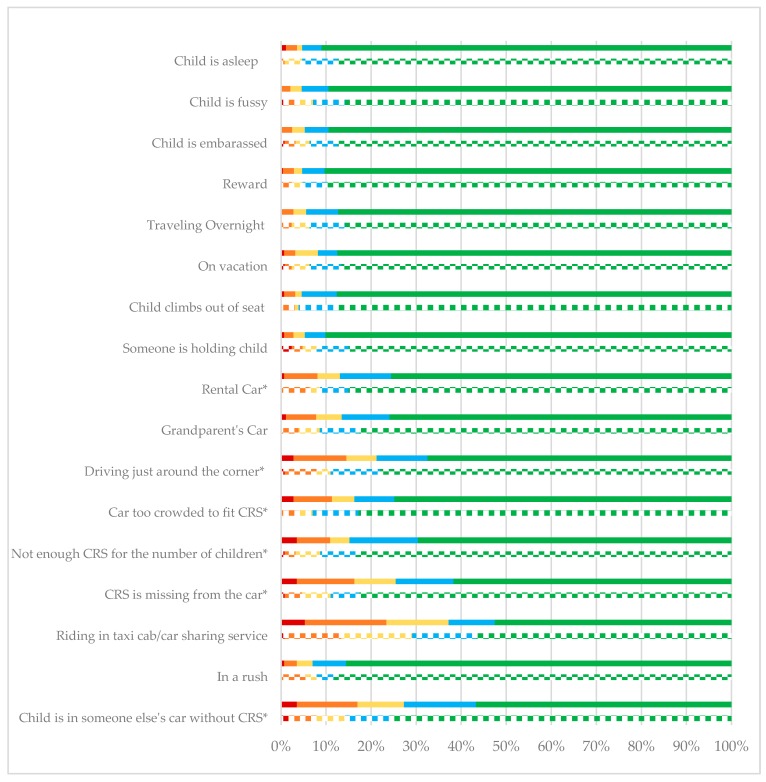

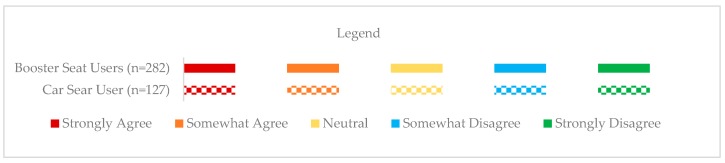
“I would allow my oldest child between ages 4–10 years to ride in a car not fully buckled in their CRS when…” Note: Significant differences across groups are noted with an * *p* < 0.05.

Figure 1 outlines both the distribution of responses for situational use of CRSs. There were significant differences (*p* < 0.05) among the participants who reported most often using booster seats and car seats in situations involving rental cars, driving just around the corner, car too crowded to fit the CRS, not enough CRSs in the vehicle, the CRS is missing from the car, or the child is in someone else’s car without a CRS (*p* < 0.05).

### 3.2. Carpooling

As outlined in Supplementary Figure S3, 519 participants most often used a booster seat, seat belt or none of the above and fell within the height of 38–56 in (96.5–142.2 cm). Of that group, 57.0% (n = 296) car pooled other children. Table 2 outlines the demographic data of the 296 participants who carpooled other children; 53.7% most often used booster seats and 46.3% reported typically using a seat belt or none of the above for their child (non-booster seat users).

As outlined in Supplementary Figure S4, of the 519 participants who most often used a booster seat, seatbelt, or none of the above and fell within the height of 38–56 in (96.5–142.2 cm), 73.2% (n = 380) of the sample had their children transported by another individual. Table 3 outlines the demographic data of the 380 participants whose child has been carpooled by others—55.8% used booster seats, and 44.2% reported typically using a seat belt or none of the above for their child (non-booster seat users).

**Figure 2:**
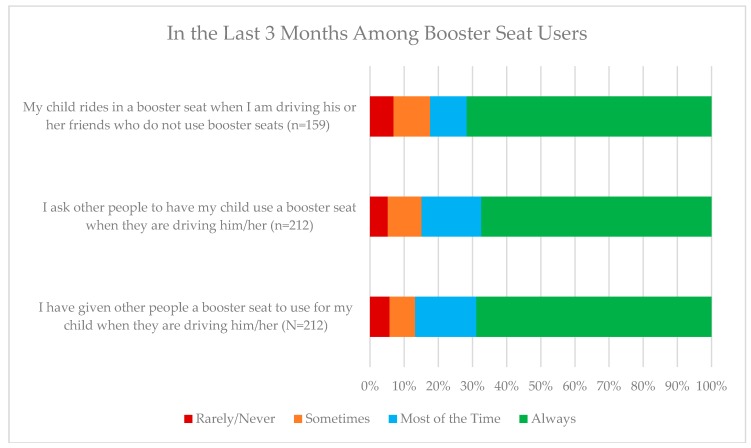
Booster seat users and their carpooling behaviors.

Figure 2 displays their reported behaviors of just participants who indicated they most often used booster seats who either carpooled other children (n = 159) or had their child transported by others (n = 212). Among booster seat users who carpooled other children (n = 159), 71.7% (n = 114) always used a booster seat for their own child, with 6.9% (n = 11) indicating they never used a booster seat. Among booster seat users who have their own child carpooled by others (n = 212), 67.5% (n = 143) indicated they always ask other people to use a booster seat for their child when driving them and a similar percentage (68.9%, n = 146) indicated they always give their booster seat to someone else when they are driving them.

**Figure 3:**
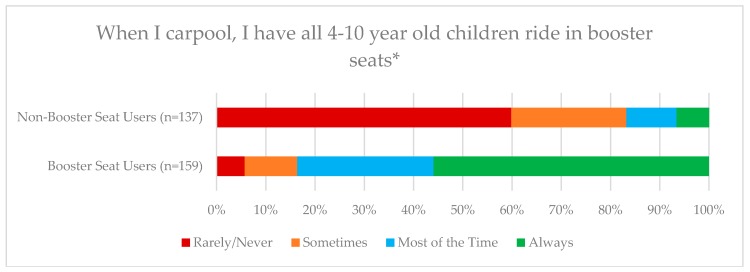
Non-Booster Seat users (seatbelts or none of the above) (137) vs. Booster Seat users (159). * *p* < 0.05 for significant differences between groups on distribution of responses.

Figure 3 compares the distribution of responses for the booster seat and non-booster seat users in response to the item of “When I carpool, I have all 4–10 years old children ride in booster seats.” There were significant differences in response among the two groups. When comparing booster-seat users to non-booster seat users, booster seat users were significantly more likely to use booster seats for other children ages 4–10 (*p* < 0.01) (Figure 3).

## 4. Discussion

This survey of parents and caregivers of children ages 4–10 years old has outlined situations in which they do no fully restrain their child in their CRS. Overall, we saw that parents/caregivers that most often used boosters were more willing than those who used car seats to not fully restrain their child due to practical issues—such as driving a close distance, too many people in the car to fit the CRS, not enough CRSs in the car, not having a CRS in the car, rental cars or being in a friend’s car that does not have a CRS. In addition to these practical situations, we saw that for carpooling, those who used booster seats for their own child were significantly more likely to use booster seats for other children than those who did not use booster seats for their own children. However, we also found that even for those who used booster seats for their own child, they were not consistently using them for other children ages 4–10 in the vehicle, asking others to use a booster seat for their own child, or providing a booster seat to others for the carpooling experience.

The differences in willingness to not fully restrain their child among booster seat and car seat users in our sample is consistent with trends in overall compliance with booster seats, as compared to car seats [7,8]. We did not evaluate the parents’ and caregivers’ risk perceptions of injury relative to CRS use and motor vehicle crashes. It would be beneficial to further evaluate the risk perceptions associated with the situational use of CRSs [22], and the differences between parents who most often use booster and car seats. Perceived benefits, decreased barriers to access, and social norms have been identified as factors associated with booster seat use [23,24]. Our results also highlight the potential influence of temporal decision-making, where present reward (i.e., getting to where they need to go) is outweighed by negative outcomes (i.e., possible child injury due to non-restraint as a result of a motor vehicle crash) [19,25]. Some of the situations described involve advanced planning (e.g., such as vacation or overnight driving) or emotional reactions of the child (e.g., being fussy) had relatively fewer parents agreeing or strongly agreeing that they would not fully buckle their child than access to CRSs. Although we did not survey parents/caregivers on the topic of cost or portability, options for parents in situations such as when they are not in their own vehicle are needed to better understand improved uptake of CRS use.

Similar to Macy and colleagues [10], we found that many parents do not always use a booster seat in carpooling situations. This previous study was conducted in 2010, prior to the American Academy of Pediatrics Child Passenger Safety Policy Statement and Technical Report in 2011 [1,2], yet even with these new guidelines, inconsistent booster seat use in carpooling still exists. Even among those who typically use booster seats, only 71.7% reported always using a booster seat for their own child when carpooling other children, and only 67.5% reported always asking others to have their child ride in a booster seat when others are driving them. The lack of booster seat use among parents/caregivers for transporting other children is also a concern. Our results highlight the importance of parents and caregivers asking others who are transporting their child what type of CRS will be used for the trip, as well as asking for a booster seat to be used for their child. Improving CRS use in carpooling behaviors may benefit from messaging associated with positive descriptive norms [26]. Further research is needed to better understand the frequency, nature and CRS use that could occur in these carpooling situations.

Technology is advancing in CRSs, as well as vehicles, but parents and caregiver need to utilize the CRS in order to keep their child safe. To support parents and caregivers in using the appropriate CRSs for their child on every ride, interventions and public health campaigns may need be targeted and have clear messages to address those in which parents may be more willing to not fully buckle their child [27,28]. For example, we saw that for the taxi and car sharing service situation, 23.6% and 13.1% of booster seat and car seat users, respectively, agreed or somewhat agreed they would not fully buckle their child. This could be a clear target area for screening and counseling by health care providers, as well as public health campaigns addressing social norms associated with booster seat use.

### Limitations

This study used self-report data from an online platform and therefore does not include direct observation of CRS use. The convenience sample was not nationally representative. As was reflected in our sample, the demographic characteristics of respondents from mTurk are often found to be more educated, less diverse and have a higher income than a nationally representative sample [18]. Thus, the survey methodology used in this study limits the generalizability of the results. We did not query individuals who were car seat users about their carpooling behaviors, and therefore cannot draw conclusions about their behaviors. We did not ask the frequency at which participants engaged in situational use, rather what their willingness would be for not fully buckling their child.

## 5. Conclusions

Decreased compliance with CRS use places children at risk for injury if there is a motor vehicle crash. There are a number of situations, in particular practical ones, in which parents and caregivers are willing to not fully restrain in their CRS. Parents and caregivers who most often used booster seats as compared to car seats for their children ages 4–10 years old, were more willing to not fully restrain their child due to practical issues. For caregivers who carpooled, those who do not use booster seats for their own child were significantly less likely to use booster seats for other children. Continued education and programs surrounding consistent and safe child restraint use is critical, particularly for children who should be in booster seats.

## Figures and Tables

**Table 1:** Demographic characteristics for situational use analysis.

Age	Total Sample (409)	Car Seat Users (127)	Booster Seat Users (282)
18–29	86 (21.0%)	39 (30.7%)	47 (16.7%)
30–39	228 (55.7%)	62 (48.8%)	166 (58.9%)
40–49	83 (20.3%)	22 (17.3%)	61 (21.6%)
50+	12 (2.9%)	4 (3.1%)	8 (2.8%)
**Race**			
White	365 (89.2%)	110 (86.6%)	255 (90.4%)
Not White	44 (10.8%)	17 (13.4%)	27 (9.6%)
**Hispanic**			
No	381 (93.2%)	118 (92.9%)	263 (93.3%)
Yes	28 (6.8%)	9 (7.1%)	19 (6.7%)
**Gender**			
Male	156 (38.1%)	50 (39.4%)	106 (37.6%)
Female	253 (61.9%)	77 (60.6%)	176 (62.4%)
**Relationship to Child**			
Mother/Step-mother/foster mother	247 (60.4%)	73 (57.5%)	174 (61.7%)
Father/Step-father/foster father	149 (36.4%)	48 (37.8%)	101 (35.8%)
Grandparent/Sibling/Other	13 (3.2%)	6 (4.7%)	7 (2.5%)
**Income**			
Less than $25,000	36 (8.8%)	11 (8.7%)	25 (8.9%)
$25,000 to $49,999	124 (30.3%)	41 (32.3%)	83 (29.4%)
$50,000 to $74,999	112 (27.4%)	34 (26.8%)	78 (27.7%)
$75,000 to $99,999	68 (16.6%)	24 (18.9%)	44 (15.6%)
$100,000+	69 (16.9%)	17 (13.4%)	52 (18.4%)

**Table 2:** Participant carpooling other children: n = 296.

Age	Total Sample (n = 296)	Booster Seat Users (n = 159)	Non Booster Seat Users (n = 137)
18–29	43 (14.5%)	28 (17.6%)	15 (10.9%)
30–39	161 (54.4%)	87 (54.7%)	74 (54.0%)
40–49	81 (27.4%)	40 (25.2%)	41 (29.9%)
50+	11 (3.7%)	4 (2.5%)	7 (5.1%)
**Race**			
White	258 (87.2%)	146 (91.8%)	112 (81.8%)
Not White	38 (12.8%)	13 (8.2%)	25 (18.2%)
**Hispanic**			
No	278 (93.9%)	146 (91.8%)	132 (96.4%)
Yes	18 (6.1%)	13 (8.2%)	5 (3.6%)
**Gender**			
Male	130 (43.9%)	68 (42.8%)	62 (45.3%)
Female	166 (56.1%)	91 (57.2%)	75 (54.7%)
**Relationship to Child**			
Mother/Step-mother/foster mother	160 (54.1%)	90 (56.6%)	70 (51.1%)
Father/Step-father/foster father	126 (42.6%)	64 (40.3%)	62 (45.3%)
Grandparent/Sibling/Other	10 (3.4%)	5 (3.2%)	(3.7%)
**Income**			
Less than $25,000	24 (8.1%)	10 (6.3%)	14 (10.2%)
$25,000 to $49,999	84 (28.4%)	49 (30.8%)	35 (25.5%)
$50,000 to $74,999	77 (26.0%)	42 (26.4%)	35 (25.5%)
$75,000 to $99,999	50 (16.9%)	26 (16.4%)	24 (17.5%)
$100,000+	61 (20.6%)	32 (20.1%)	29 (21.2%)

**Table 3:** Participant’s child being carpooled by another person n = 380.

Age	Total Sample (n = 380)	Booster Seat Users (n = 212)	Non Booster Seat Users (n = 168)
18–29	56 (14.7%)	38 (17.9%)	18 (10.7%)
30–39	210 (55.3%)	121 (57.1%)	89 (53.0%)
40–49	96 (25.3%)	47 (22.2%)	49 (29.2%)
50+	18 (4.7%)	6 (2.8%)	12 (7.1%)
**Race**			
White	330 (86.8%)	189 (89.2%)	141 (83.9%)
Not White	50 (13.2%)	23 (10.8%)	27 (16.1%)
**Hispanic**			
No	357 (93.9%)	197 (92.9%)	160 (95.2%)
Yes	23 (6.1%)	15 (7.1%)	8 (4.8%)
**Gender**			
Male	164 (43.2%)	84 (39.6%)	80 (47.6%)
Female	216 (56.8%)	128 (60.4%)	88 (52.4%0
**Relationship to Child**			
Mother/Step-mother/foster mother	208 (54.7%)	127 (59.9%)	81 (48.2%)
Father/Step-father/foster father	161 (42.4%)	80 (37.7%)	81 (48.2%)
Grandparent/Sibling/Other	11 (3.9%)	5 (2.3%)	6 (3.6%)
**Income**			
Less than $25,000	39 (10.3%)	20 (9.4%)	19 (11.3%)
$25,000 to $49,999	102 (26.8%)	59 (27.8%)	43 (25.6%)
$50,000 to $74,999	107 (28.2%)	58 (27.4%)	49 (29.2%)
$75,000 to $99,999	60 (15.8%)	36 (17.0%)	24 (14.3%)
$100,000+	71 (18.7%)	39 (18.4%)	32 (19.0%)

## Supplementary Materials

The following are available online at http://www.mdpi.com/1660-4601/15/8/1788/s1, Figure S1: Overall Analytic Sample n = 783; Figure S2: Situational Use Sample n = 409; Figure S3: Participant Carpooling Other Children: n = 296; Figure S4: Participant’s Child being Carpooled by Another Person n = 380.
