# Supplementary material for: Situational Use of Child Restraint Systems and Carpooling Behaviors in Parents and Caregivers
Source: Int J Environ Res Public Health. 2018 Aug 20;15(8):1788. doi: 10.3390/ijerph15081788 (PMC6121359; doi:10.3390/ijerph15081788)

Figure S1: Overall Analytic Sample n=783

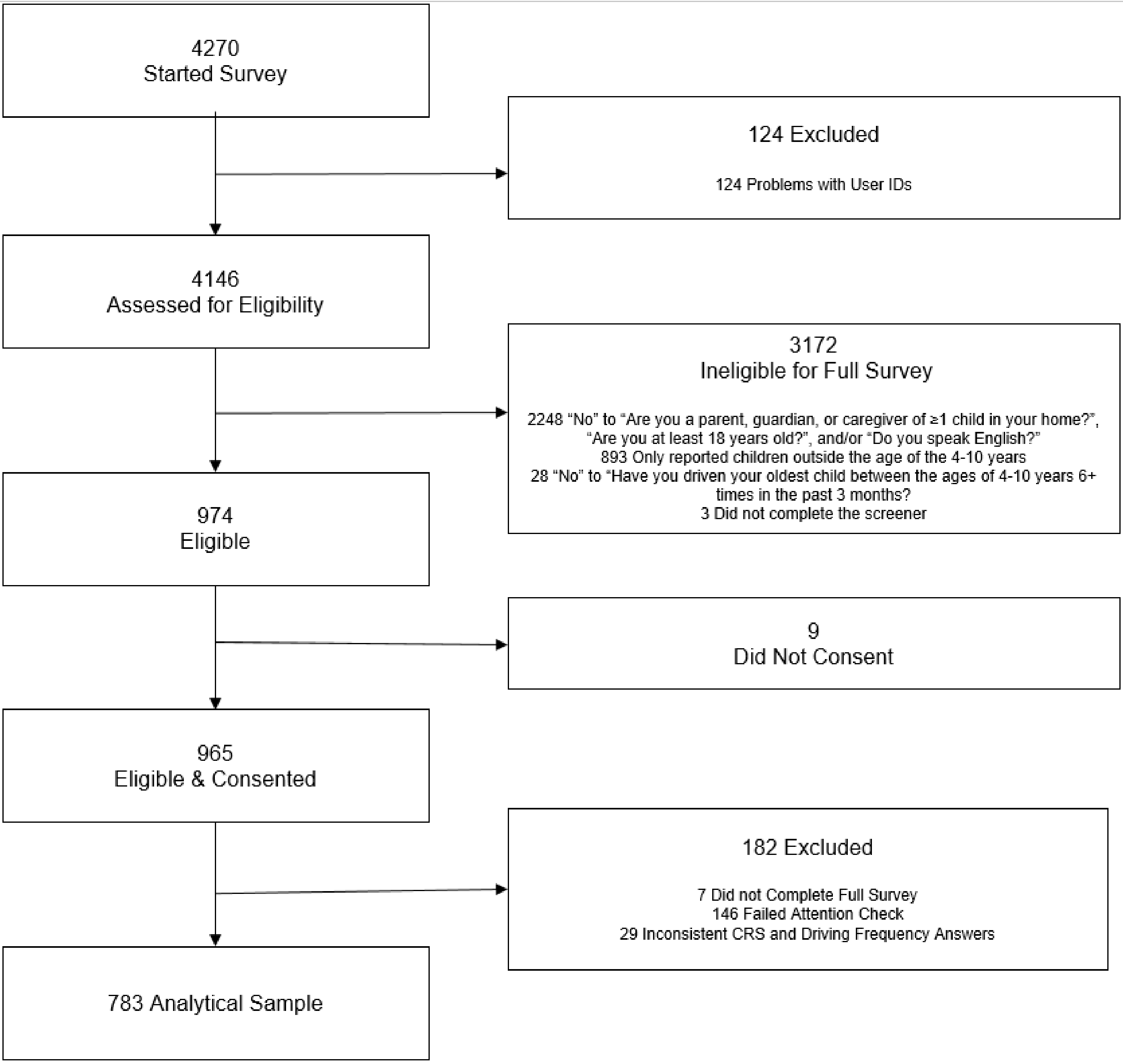

Figure S2: Situational Use Sample n=409

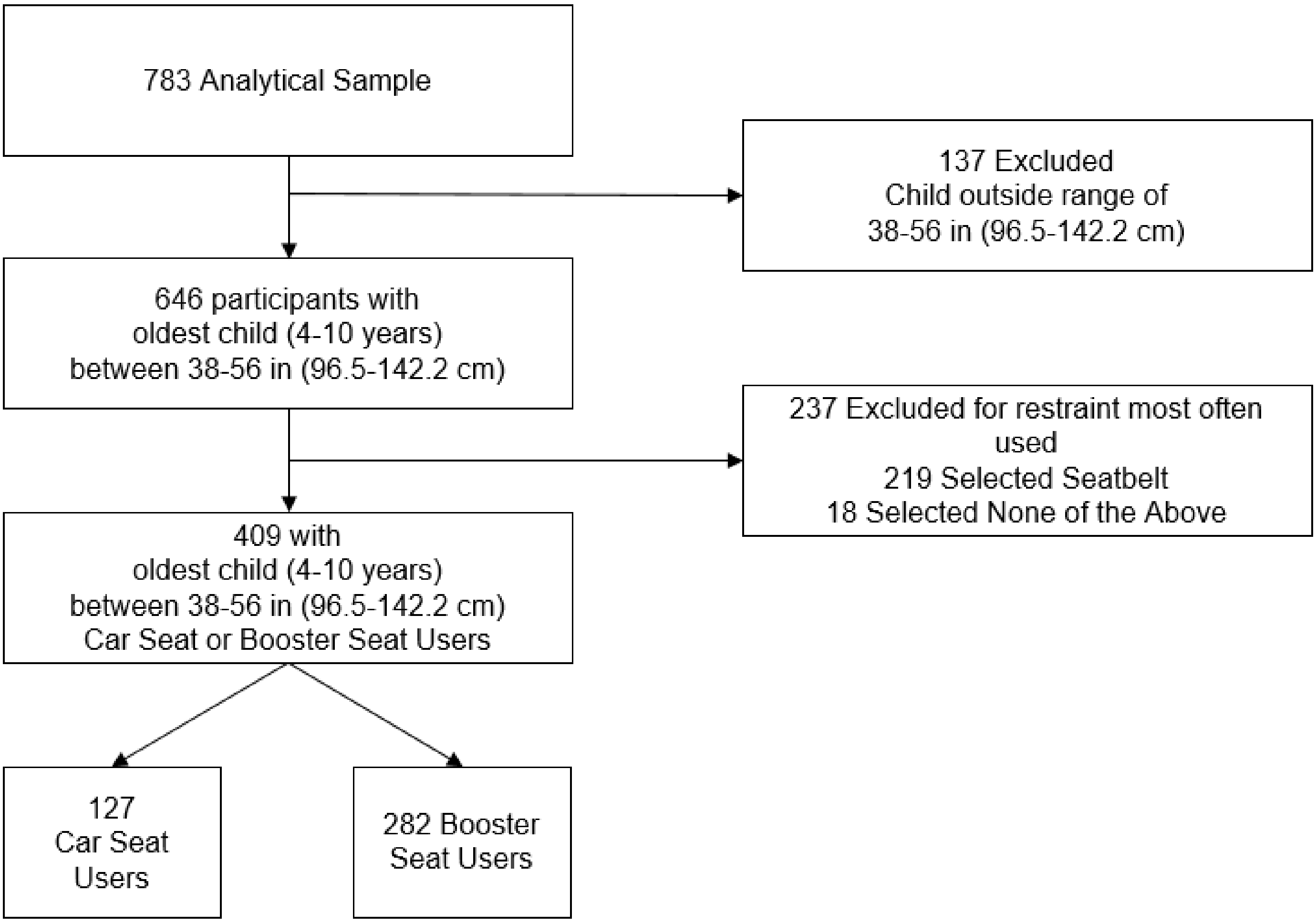

Figure S3: Participant Carpooling Other Children: N=296

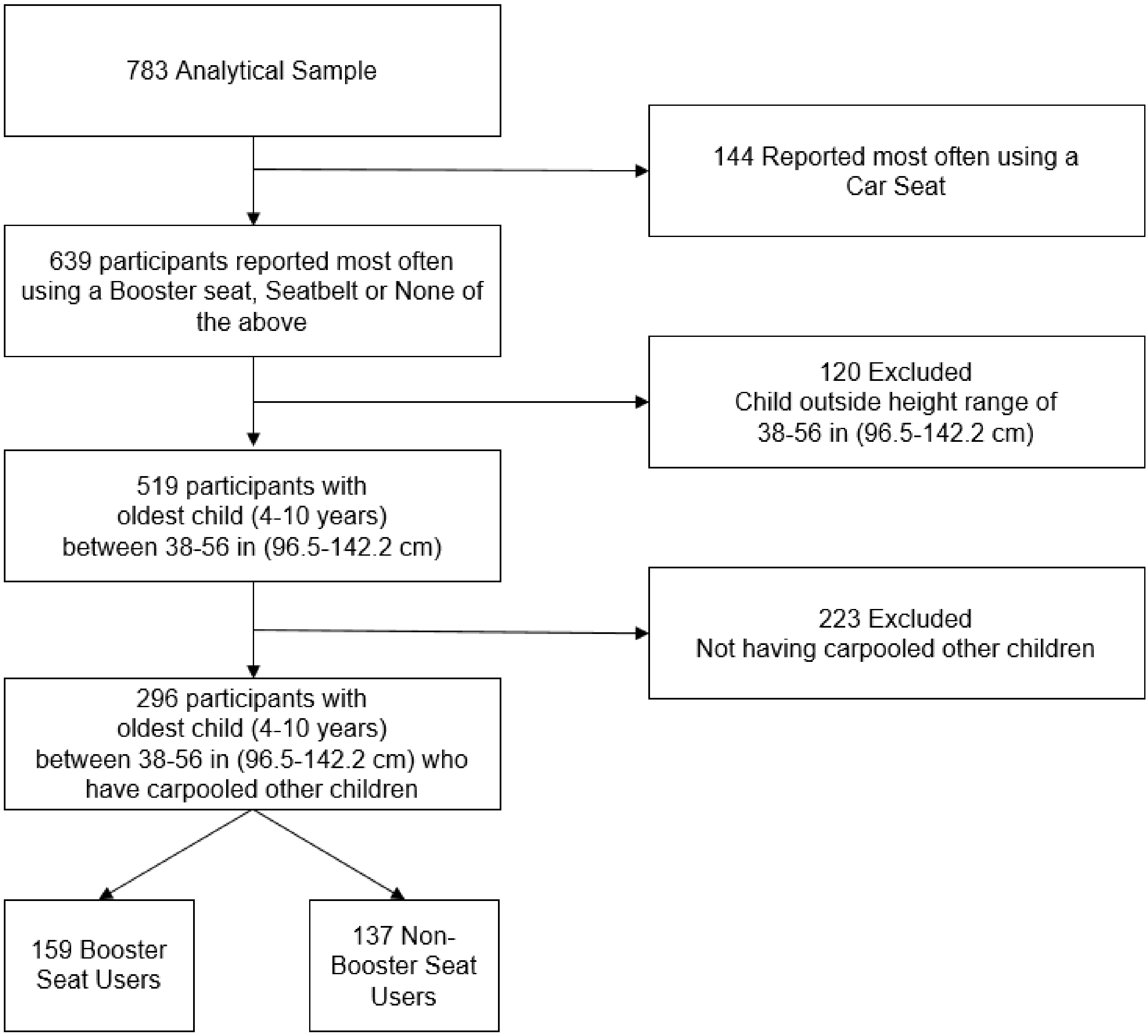

Figure S4: Participant's Child being Carpooled by Another Person n=380

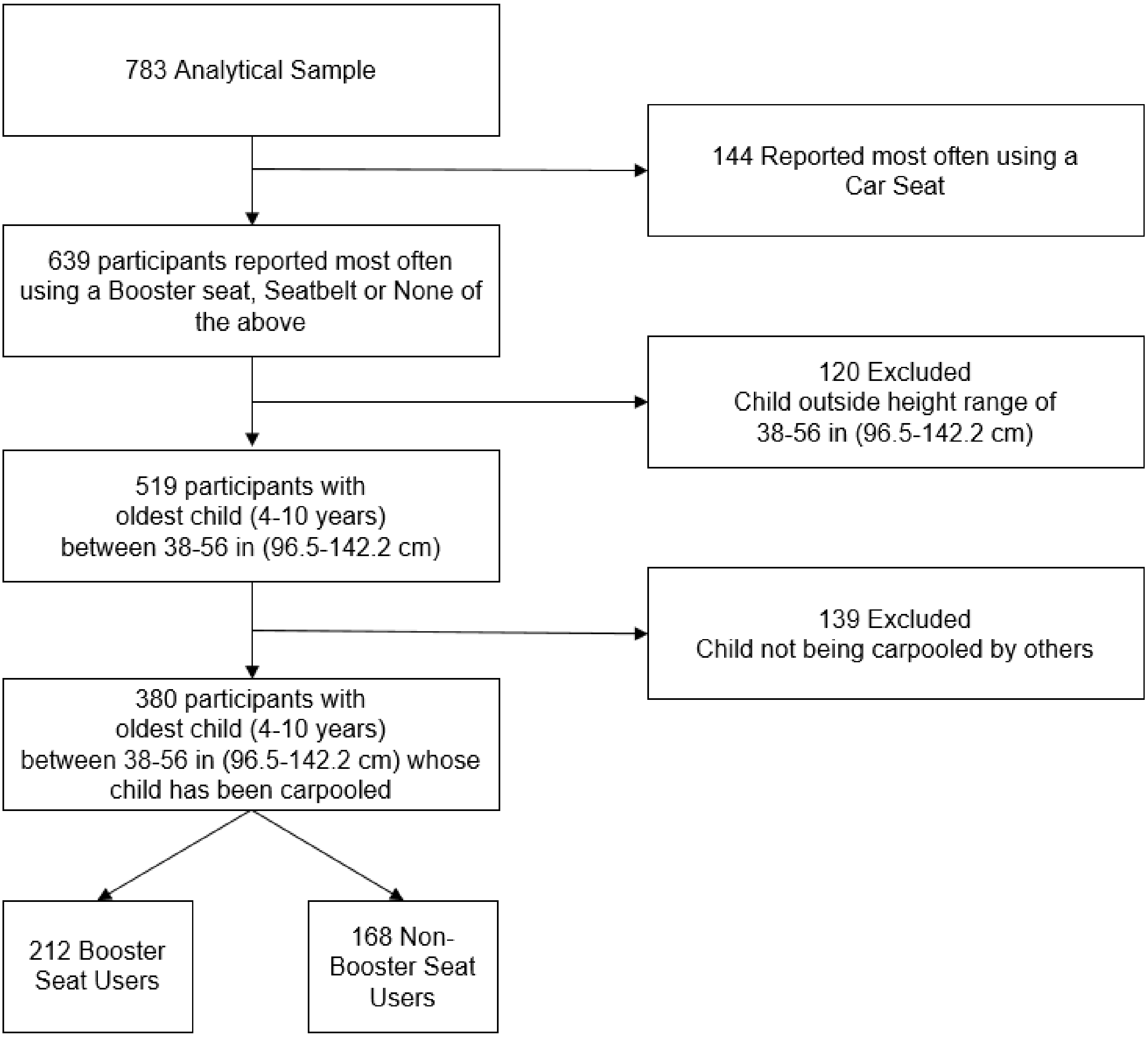

Supplement: Supplementary file 1 [file ijerph-15-01788-s001.pdf]
